# The role of innate immunity in the protection conferred by a bacterial infection against cancer: study of an invertebrate model

**DOI:** 10.1038/s41598-020-66813-0

**Published:** 2020-06-22

**Authors:** Camille Jacqueline, Jean-Philippe Parvy, Marie-Lou Rollin, Dominique Faugère, François Renaud, Dorothée Missé, Frédéric Thomas, Benjamin Roche

**Affiliations:** 1 CREEC, MIVEGEC, UMR IRD/CNRS/UM 5290, 911 Avenue Agropolis, BP 64501, 34394 Montpellier Cedex 5, France; 2 Department of Immunology, School of Medicine, University of Pittsburgh, Pittsburgh, PA 15261 USA; 3 CRUK Beatson Institute, Institute of Cancer Sciences, University of Glasgow, Garscube Estate, Switchback Road, Glasgow, G61 1BD UK; 4 International Center for Mathematical and Computational Modeling of Complex Systems (UMI IRD/UPMC UMMISCO), 32 Avenue Henri Varagnat, 93143 Bondy Cedex, France; 5 Departamento de Etología, Fauna Silvestre y Animales de Laboratorio, Facultad de Medicina Veterinaria y Zootecnia, Universidad Nacional Autónoma de México (UNAM), Ciudad de Mexico, Mexico

**Keywords:** Cancer models, Infection, Innate immunity

## Abstract

All multicellular organisms are exposed to a diversity of infectious agents and to the emergence and proliferation of malignant cells. The protection conferred by some infections against cancer has been recently linked to the production of acquired immunity effectors such as antibodies. However, the evolution of innate immunity as a mechanism to prevent cancer and how it is jeopardized by infections remain poorly investigated. Here, we explored this question by performing experimental infections in two genetically modified invertebrate models (*Drosophila melanogaster*) that develop invasive or non-invasive neoplastic brain tumors. After quantifying tumor size and antimicrobial peptide gene expression, we found that *Drosophila* larvae infected with a naturally occurring bacterium had smaller tumors compared to controls and to fungus-infected larvae. This was associated with the upregulation of genes encoding two antimicrobial peptides—diptericin and drosomycin—that are known to be important mediators of tumor cell death. We further confirmed that tumor regression upon infection was associated with an increase in tumor cell death. Thus, our study suggests that infection could have a protective role through the production of antimicrobial peptides that increase tumor cell death. Finally, our study highlights the need to understand the role of innate immune effectors in the complex interactions between infections and cancer cell communities in order to develop innovative cancer treatment strategies.

## Introduction

The bodies of virtually all multicellular organisms can be considered as holobionts that are composed—in addition to host cells—of a diversity of living entities, including microbiota, parasites, and malignant cells^1^. While reciprocal interactions between host phenotype and symbiotic organisms have long fascinated evolutionary ecologists^2–6^, oncogenic phenomena have scarcely been considered as potential players in these interactions^7,8^. Nevertheless, malignant cells have been omnipresent in the bodies of multicellular organisms for more than half a billion years^9^, and not only in post-reproductive stages^10^. They are furthermore involved in reciprocal interactions with microbes and parasites^11^, hence setting the scene for fascinating, yet complex, tripartite interactions. Understanding these interactions is an emerging and relevant topic in ecology and evolution since any phenotypic change related to health, however small, can be associated in the wild with a higher risk of predation and/or infection as well as reduced competitiveness/attractiveness in sexual selection processes^12^.

The indirect role of infections on tumor dynamics has been recently reviewed, highlighting the importance of immune responses in explaining the protection conferred by certain infectious agents^13^. Indeed, numerous immune mechanisms are shared by anti-infection and anti-cancer responses^14^, and the impact of infectious agents on tumoral immunity has been studied in mammal models. For instance, tumor regression following Bacille Calmette Guerin (BCG) vaccination has been linked with the infiltration of BCG-specific T cells into the tumor^15^. In addition, it has been shown that influenza-experienced mice control lung tumor challenge better than naïve mice through the generation of antibodies specific to the antigens shared by both infected and cancer cells^16^. Epidemiological studies have shown that antibodies against tumor-associated antigens can be found in patients who experienced a large number of acute infectious or inflammatory events early in life and who had a drastically reduced risk of ovarian cancer later in life^17–19^.

We see that the role of adaptive immunity, specific to infectious antigens and providing long-lasting responses, has been well-described in terms of cancer protection conferred by exposure to infections. However, early work by William Coley^20^ as well as recent reports in mice^21^ have unambiguously suggested that innate immunity, i.e., a non-specific defense that comes into play immediately or within hours of an antigen’s appearance, may be particularly relevant when trying to understand the interactions between infections and cancer^21–23^. Because it is technically challenging to create mammal models totally deprived of adaptive effectors, the use of an invertebrate model that does not evolve adaptive immunity (but see^24,25^ for evidence of immune memory) could bring new and relevant findings to this compelling topic. Since neoplastic lesions have been reported in a wide range of invertebrates from hydra to starfish^26,27^, and because those organisms only harbor innate immune effectors, natural selection may have selected them to eliminate aberrant cells. Nevertheless, the role of infectious pressures on tumor development in invertebrates has been poorly investigated, with the exception of studies on infectious neoplasia^28^.

To disentangle the role of infection-induced innate immunity in tumor burden, we thus used *Drosophila*, which is recognized as a pertinent model for the study of cancer^29^. In *Drosophila*, the production of antimicrobial peptides (AMP) is an important aspect of host defense. They are synthetized by the fat body (a multifunctional tissue involved in immune responses, energy storage, and nutritional sensing) in response to infections and circulate in the blood. These peptides can be grouped into three families based on their main biological targets, i.e., gram-positive bacteria (defensin), gram-negative bacteria (cecropins, drosocin, attacins, diptericin), or fungi (drosomycin, metchnikowin)^30^. Another type of host defense mechanism to injury, parasitoid wasp infection, or aberrant cells is the activation of the Janus Kinase/Signal Transducer and Activator of Transcription (Jak/STAT) pathway^31–33^. Interestingly, Jak-STAT has been shown to regulate AMP production in response to damage and stress but not microbial products^34^. Thus, we carried out two types of acute infection, one with a gram-negative bacterium and the other with a fungus, on an experimental model that develops GFP-labeled invasive neoplastic tumors in a larval epithelial tissue called the eye-antennal disc^35^. Extensive image analysis of the tumors and transcriptomic study of two main AMP genes and of an activator of the Jak-STAT pathway allowed us to hypothesize the role of infection in tumor regression. Using an independent tumor model, we then confirmed tumor regression following bacterial infection and suggest that this decrease is, at least in part, due to an increase of tumor cell death that could be mediated by the AMP production.

## Results

### Innate immune responses to tumor cells and impact of infections on tumor size

**Figure 1:**
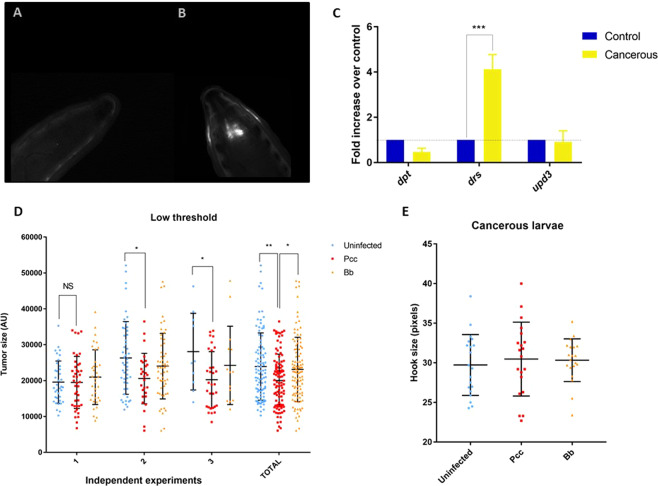
Tumor visualization *in vivo* in (**A**) non-cancerous and (**B**) cancerous larvae characterized by GFP-labeled cells (in white) in the eye-antennal disc region. (**C**) Fold induction of the three immune genes in cancerous larvae (yellow; n = 10 pools) without infection relative to non-cancerous larvae (dark blue; n = 10 pools). The error bars represent standard deviation (SD) of two independent experiments (***p < 0.001; **p < 0.01; *p < 0.05; Sidak’s test). Effect of infection by the bacterium *Pectobacterium carotovorum carotovorum* (*Pcc*, red; n = 96 total) and the fungi *Beauvaria bassiana* (*Bb*, orange; n = 102 total) compared to uninfected flies (blue; n = 97 total) on individual tumor size with a low intensity threshold of detection (S2 threshold: 75% of maximum intensity value) in three independent experiments. The error bars represent standard deviation (***p < 0.001; **p < 0.01; *p < 0.05; NS > 0.05; Tukey’s test). (**E**) Impact of body size, approximated by mouth hook size, on tumor size depending on infectious treatment (n = 20 by infection group; error bars are SD).

Our *scrib*^1^*/Ras*^*V*1*2*^ mutant model allows visualization of the tumor *in vivo* in late 3^rd^ instar larvae using a dissecting microscope under GFP fluorescence (Fig. 1A,B). We first measured activation of the innate immunity in non-infected cancerous larvae. qRT-PCR analyses revealed that *scrib*^1^*/Ras*^*V*1*2*^ mutants showed higher levels of *drosomycin* (*drs*) gene expression than non-cancerous larvae (Fig. 1C; p = 0.0003). However, we did not find increased gene expression of *diptericin* (*dpt*) or of the cytokine *unpaired 3* (*upd3*, a key activator of the Jak/STAT pathway). This suggests that the presence of tumor cells could induce activation of the *drs* gene in the fat body or in the circulating hemocytes.

To test whether *drs* activation was a protective mechanism against cancer, we measured the effect of different infectious treatments on tumor size in our *Drosophila* model. We compared tumor growth in non-infected larvae and in larvae infected by the gram-negative bacterium *Pectobacterium carotovorum carotovorum* (*Pcc*) or by the fungal entomopathogen *Beauvaria bassiana* (*Bb*), both known to strongly stimulate AMP production^36^. Analysis of tumor phenotype showed that larvae orally infected by the *Pcc* bacterium showed a 20% decrease in tumor size compared to uninfected ones (Fig. 1D; p = 0.004) and *Bb* infected ones (p = 0.026). This was also true when considering a more restricted threshold of detection (Figure S1; Supplementary materials). Infection by *Bb* did not significantly affect tumor size compared to controls. We also confirmed that the dose used to induce infections did not cause a delay in development, which could impact tumor size, and showed that the body size of infected larvae is similar to that of uninfected larvae (Fig. 1E). In addition, the lack of correlation between body size and tumor size in bacterial-infected larvae suggests that cancerous larvae with large tumors were not more susceptible to the infection (Figure S2, Supplementary materials).

We therefore further analyzed AMP production following bacterial infection in an attempt to explain its specific impact on tumor size, looking for mechanisms that would not occur with a fungal infection.

### Innate immune responses to infections and function of dpt in tumor regression

**Figure 2:**
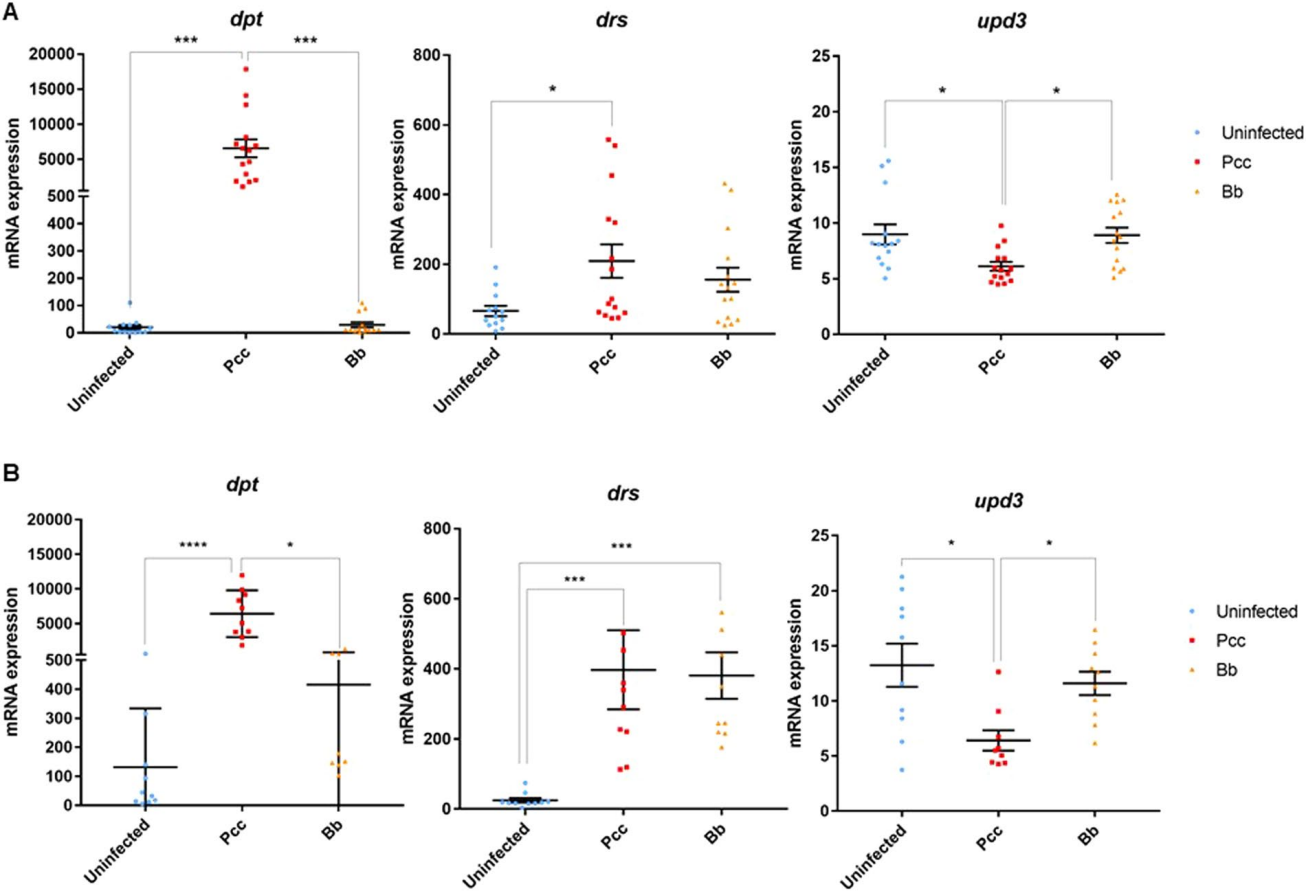
(**A**) mRNA levels of expression for the three AMP genes, *dpt*, *drs*, and *upd3*, in cancerous larvae in response to infection by the bacterium *Pcc* (red; n = 15 pools) and the fungi *Bb* (orange; n = 14 pools) compared to uninfected larvae (Ctl, blue; n = 13 pools). Data shown here were pooled from five independent experiments (***p < 0.001; **p < 0.01; *p < 0.05; Kruskal-Wallis test). Gene expression was assessed by qRT PCR with the primers described in Table S2. (**B**) mRNA levels of expression for the three AMP genes (*dpt*, *drs, upd3*) in non-cancerous larvae following infections (n = 10 pools by infection group). The error bars represent standard deviation and data were pooled from two independent experiments (****p < 0.0001; ***p < 0.001; **<0.01; *p < 0.05; Kruskal-Wallis test).

To identify which AMP plays a role in the specific effect of bacterial infection on tumor regression, we studied the upregulation of AMP genes following infections in cancerous larvae. On the one hand, infection with *Pcc* resulted in a 50-fold increase of *dpt* mRNA expression compared to non-infected larvae (Fig. 2A; p < 0.0001) as well as in an increase of *drs* expression (p = 0.024). The same analyses were conducted in non-cancerous larvae, and we found that the bacterial infection also upregulated *dpt* gene expression (Fig. 2B; p < 0.0001). However, contrary to what was observed in cancerous larvae, *drs* gene expression was found to be upregulated not only following bacterial infection (p = 0.006) but also following fungal infection (Fig. 2B; p = 0.003).

To determine whether AMP production could result from cell damage that was induced either by the infection or by immune responses (in a manner independent of microbial products), we measured gene expression of cytokine *upd3* and found that it was downregulated in bacterial-infected cancerous larvae (Fig. 2A; p = 0.016) and non-cancerous larvae (Fig. 2B; p = 0.014). All together, these data indicate that microbial products from *Pcc* activate the production of *dpt* and *drs*, which could increase tumor cell death and thus be involved in tumor regression.

### Tumor cell death and regression following bacterial infection in dlg^40.2^ mutants

**Figure 3:**
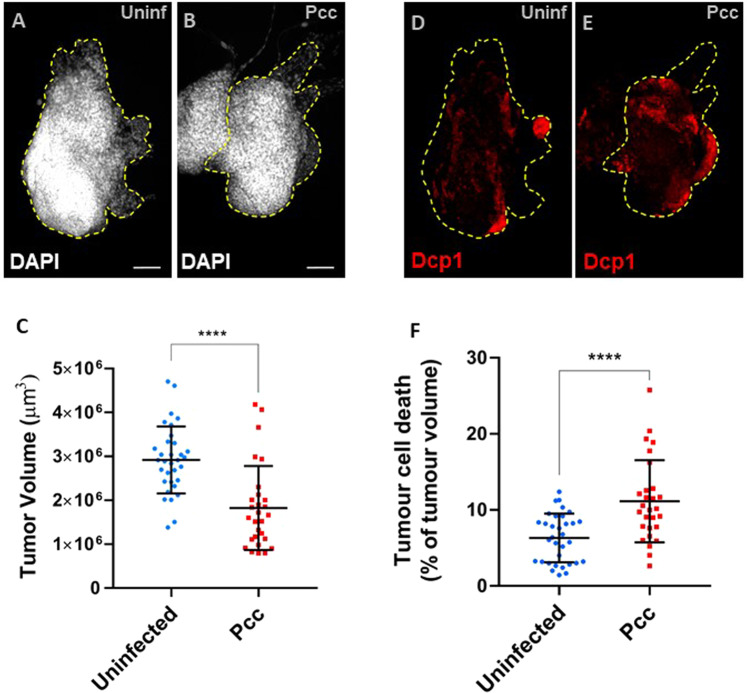
(**A,B**) Representative immunofluorescence images of the tissues quantified in both conditions, stained with DAPI (white) to measure tumor volume. Wing discs are highlighted by the yellow dotted lines. Scale bars = 50 mm. (**C**) Quantification of wing disc tumor volumes from uninfected *dlg* mutant larvae (Uninfected, n = 33) or from *dlg* mutant larvae infected with *Pcc* (n = 28). (**D,E**) Representative immunofluorescence images of the quantified tissues that have been stained with the anti-Dcp1 antibody (red) to measure tumor cell death. F) Quantification of wing disc tumor cell death  in *dlg* mutant larvae uninfected (Uninfected, n = 33) or infected with *Pcc* (n = 28). Results are presented as a pool of two independent replicates (****p < 0.0001; Student’s t-test).

To further confirm that bacterial infection with *Pcc* and the associated AMP production can trigger tumor regression, we made use of *dlg*^*40.2*^ mutants (hereafter referred to as *dlg*), an independent tumor model leading to the formation of non-invasive neoplastic tumors in the wing disc. Using tumor dissection, immunostaining, and confocal imaging (Fig. 3A,B), we observed that oral infection of *dlg*-mutant larvae with *Pcc* led to strong decreases in tumor volume, reaching roughly 38% (Fig. 3C; p < 0.0001). Interestingly, using anti-dcp1 staining as a marker of tumor cell death (Fig. 3D,E), we observed a 76% increase in caspase activation (Fig. 3F; p < 0.0001). These results confirm that *Pcc* infection is a robust inducer of tumor regression at least in part through an increase in tumor cell death.

## Discussion

Multicellular organisms are not autonomous entities, but rather “holobionts” composed of the host cells, including those carrying oncogenic mutations, plus all of its commensal and mutualistic microorganisms as well as a diversity of more or less prevalent parasite taxa (viruses, bacteria, fungi, protozoa, and metazoans)^1^. However, triple reciprocal interactions involving malignant cells, infections, and hosts have been until now little studied. In this context, our study demonstrates that naturally occurring bacterial infection can promote tumor regression in two drosophila tumor models, and it supports the idea that infection-dependent production of diptericin and drosomycin, two antimicrobial peptides, may be key drivers of this regression through increased tumor cell death. Our results are supported by previous studies showing that increase in Toll and Imd signaling in tissues distant from the tumor result in increased tumor cell death in *dlg* mutant animals^37–39^. Imd and Toll pathways could act synergistically to produce AMP, as already reported elsewhere^40^. Even if *dpt* and *drs* are considered as read-outs of the Imd and Toll pathways, respectively, further studies are needed to assess the regulation of pathway-specific genes such as Dif, Dorsal, and Relish and to confirm the involvement of their cross-activation in bacterial-induced tumor regression. In addition, we cannot exclude that other mechanisms—such as the tumor’s intrinsic activation of immune pathways, which are reported to play a role in cell competition processes^41,42^, or metabolic and/or microbiota alterations consequent to digestive infection—could have also contributed to tumor size reduction. Finally, a potential bias could be that larvae with larger tumors experience higher mortality, therefore artificially reducing tumor size. However, our observations do not support this, as we were able to show that infection by both pathogens and tumor size did not affect body condition (Figure S2).

In *scrib*^1^*/Ras*^*V*1*2*^ mutants, a local *upd3* upregulation has been observed at the tumor site in cancer cells as a response to tissue damage^31^ that can also promote tumor growth^43^. However, our data suggest that this local response is not amplified by the expression of unpaired cytokines in the fat body or the circulating hemocytes. The downregulation of *upd3* cytokine expression following the infection could represent a negative feedback loop to limit the damage associated with the infections. Another interesting result is the absence of tumor regression following fungal infection. Fungal infection is generally associated with the production of *drs*^36^, as observed in our non-cancerous individuals; however, *drs* expression was not significantly upregulated in cancerous mutants. Because *drs* was high in mutants, it could have impaired the infection. Furthermore, the absence of *dpt* upregulation could also explain the absence of tumor regression following fungal infection.

This last result indicates that protection against cancer may be conferred only by certain infections, even if innate immune effectors are thought to be mainly non-specific. It is likely that the protective mechanisms observed in response to *Pcc* infection will also be induced by other bacteria since they share common antigens called pathogen-associated molecular patterns (PAMPs)^44^. Using the *scrib*^1^*/Ras*^*V*1*2*^ model, we have been able to show that a single bacterial infection can reduce tumor size by at least 20%. However, previous work on mice has showed that two immune challenges with influenza viruses could result in a larger reduction in tumor size^16^. More generally, reports from Coley’s experiments as well as the use of bacillus Calmette-Guerin to treat superficial bladder cancer have indicated the importance of recurrent infections in promoting efficient tumor regression^20,45^. Because it is experimentally challenging to do repeated infections on a short-lived model like *Drosophila* larvae, further studies should investigate their impact on innate immunity since it could jeopardize natural anti-tumoral responses *in vivo* in long-living mammalian models.

Because humans, like all organisms, face complex schemes of infections^46^, consideration of the personal history of infections in a clinical setting could reveal relevant information for identifying individuals at risk for cancer. During the 20^th^ century, radiotherapy and chemotherapy were intensively developed to the detriment of early attempts at immunotherapy, such as Coley’s toxin^22^. However, the striking rise in immunotherapeutic strategies over the few past decades, exemplified by the attribution of the 2018 Nobel Prize in Medicine, calls for a re-evaluation of mechanisms linking infections and spontaneous tumor regression. While studies have mainly focused on adaptive immunity for the elaboration of new drugs^47^, our study, together with other recent studies in flies^38,39^, has revealed some molecular aspects mediating the forgotten potential of the innate immune system in the control of tumor burden. Further efforts should be made to elucidate the precise molecular mechanisms leading to cancer regression in response to innate immune system activation: this may also help with the design of new therapeutic strategies using drugs that mimic the effect of infections to establish an innate immune profile adverse to cancer cells. Our results may also stimulate further studies to explore the links between cancer and infections in the wild and how public health strategies targeting transmissible diseases may affect the incidence of cancer.

## Materials and Methods

### Tumor model and drosophila stocks

The genetically modified *Drosophila melanogaster* strain known as *scrib*^1^*/Ras*^*v*1*2*^ was engineered to develop a tumor of the eye-antennal discs, as previously described elsewhere^35^. Briefly, the genetic scheme uses a truncated *eyeless* promoter-driven FLP recombinase expression (*ey(3.5)-FLP*) to generate discrete patches of GFP-labeled mutant cells specifically in the developing larval eye-antennal discs. Clones are mutant for the cell polarity regulator *scribble* (*scrib*) and mis-express a constitutive active form of the ras oncogene (*Ras*^*v*1*2*^). Male *yw;* Sp/Cyo; *FRT82B/TM6* flies were crossed with *yw, ey(3.5)-FLP; act5* > *stop* > *gal4,UAS-GFP; FRT82B, Tub-gal80* females to generate non-cancerous larvae (referred to as NC) bearing GFP-labeled healthy clones. Male *UAS-Ras85D*^*v12*^*; FRT82B, scrib*^*1*^ */TM6* were crossed with the same females described above to generate cancerous larvae (referred as C) bearing *scrib*^*1*^*/Ras*^*v12*^ GFP-labeled tumors. These crosses were allowed to lay eggs on sugar-agar plates for less than 24 h, and collected embryos were incubated for two days at 24 °C. From the two crosses, we selected larvae at the late 2^nd^ or early 3^rd^ instar stage based on the lack of the dominant Tubby phenotype carried on the *TM6* balancer chromosome. The other fly stock used in this study was the *dlg*^*40.2*^*/FM7-gfp* mutant line^48^. All drosophila stocks and crosses were maintained on standard fly medium under 12 h :12 h light: dark cycles.

### Infectious agents

*Pectobacterium carotovora carotovora Ecc15* (*Pcc*) is a gram negative bacterium that can naturally infect drosophila^49^; *Pcc* used in our study was provided by the Pasteur Institut (Reference CIP 82.83 T). It was maintained in shaking culture in LB medium at 29 °C. *Beauvaria bassiana* (*Bb*) is a naturally occurring insect fungus that infects flies during sporulation. *Bb* was kindly provided by Anna Dostalova (École Polytechnique Fédérale de Lausanne, Switzerland). The fungus was grown on malt agar plates at 29 °C in the dark for two weeks.

### Infecting experiments

For the infection of *scrib*^*1*^*/Ras*^*v12*^, an overnight culture of *Pcc* was pelleted by centrifugation (15 min at 4500 rpm) and adjusted to OD_600_ = 200. Infection was done for 4 h at 29 °C by incubating larvae with 1.5 mL of bacterial suspension mixed with crushed banana in small petri dishes according to the protocol described elsewhere^49^. For *Bb* infections, *scrib*^1^/*Ras*^*v*12^ larvae were rolled on spores and then incubated in spores for 2 h at 29 °C^36,50^. Larvae were then transferred to crushed banana to avoid a bias caused by the diet change and incubated an additional 2 h at 29 °C. These two methods are expected to result in both cutaneous and oral infections for *Pcc* and *Bb* and therefore maximize the success of infection while mimicking the natural way infection occurs. Finally, a *scrib*^*1*^*/Ras*^*v12*^ control group was incubated with crushed banana with no infectious agent for 4 h at 29 °C. After incubation, seven larvae were transferred into each well of a 96-well plate filled with yeast-sugar-agar medium and wells were plugged. The persistence of the infection was assessed in random groups of larvae (without selection for cancerous status) through classical PCR with specific primers (Table S1).

For *Pcc* infection of *dlg* mutant larvae, the pelleted bacterial culture was adjusted to OD_600_ = 200 and then mixed 1:1 with a 5% sucrose solution. To set up infectious conditions, 750 μL serial dilutions (1:2 to 1:32) of bacterial solution were plated directly into bottles containing fly food, and 200 newly eclosed first instar larvae were transferred to the medium. We determined that a 1:16 dilution was the highest concentration that did not disrupt larval development (larval size and developmental delay). Larvae were grown for seven days at 25 °C and used for tumor analysis. Uninfected larvae were raised on penicillin/streptomycin containing food to avoid any bacterial contamination.

### Quantification of tumor size

For *scrib*^*1*^*/Ras*^*v12*^, we performed tumor visualization of late 3^rd^ instar larvae two days post-infection using a dissecting microscope under GFP fluorescence (Zeiss A Lovert 200 M, 2.5×). Tumors were isolated with ImageJ 1.41.0 software^51^. The intensity values of each pixel were standardized by exposure time (see Supplementary materials for more detail). Finally, tumor size was quantified with Matlab (MATLAB 2015b, The MathWorks, Natick, MA) as the number of pixels with the scaled intensity above a given threshold. Four intensity thresholds were considered to measure tumor size: pixel intensities greater than 50% (S1), 75% (S2), 90% (S3), or 95% (S4) of the maximal intensity recorded in the picture (Figure S3). Thus, we obtained four distinct tumor measurements standardized by exposure time that were analyzed separately to quantify the robustness of our results (see Supplementary materials for sensitivity on this measure).

For *dlg* mutant larvae, tumors were dissected in PBS seven days after eclosion, fixed in 4% formaldehyde, and immunostained with 4′,6-diamidine-20-phenylindole dihydrochloride (DAPI) (sigma) and rabbit anti-cleaved decapping protein 1 (Dcp1) (Cell Signaling Technology) antibody as previously described^38^. Tissues were imaged at optimal slice parameters and 12-bit resolution using a Zeiss 710 confocal microscope. We then used Volocity 3D imaging analysis software (Perkin Elmer) to quantify total tumor volume identified by DAPI staining and cell death visualized with anti-Dcp1 staining, as described in^38^.

### qRT-PCR analysis

After infections and determination of cancerous status, we obtained 10 pools of seven larvae for each group (non-infected, *Pcc-* or *Bb*-infected larvae) for non-cancerous larvae. We obtained 15 pools of seven larvae for each infectious treatment for cancerous larvae. All pools were fixed in liquid nitrogen and stored at −80 °C. Total RNA was extracted using a TRIzol reagent following the manufacturer’s protocol. Total RNA was eluted in 50 µL of water and 25 µL were used to perform DNase treatment (Turbo DNA-free kit, Life Technologies). Finally, total RNA was quantified using Nanodrop (ThermoScientific) and cDNA was synthetized from 2.5 µg of total RNA using a SuperScript III kit (SS III First-Strand Synthesis System, Life Technologies) in 20 µL total volume according to the manufacturer’s instructions.

We quantified the expression levels of the *drosomycin* (*drs*), *diptericin* (*dpt*), and *unpaired 3* (*upd3*) genes using gene-specific primers (see Table S2). PCR reactions were conducted in a total volume of 3 µL with LightCycle ® 480 SYBR Green I Master (Roche). A 10 min pre-incubation at 95 °C was followed by 40 cycles of amplification: 10 s at 95 °C, 10 s at 64 °C, and 10 s at 72 °C. Melting curves were generated after the final amplification cycle by denaturing the amplicons at 95 °C for 5 s, cooling to 65 °C for 1 min, and then increasing to 97 °C. Melting curves were used to estimate the specific melting temperature for each reaction. Each sample (*i.e*., pool) was analyzed in triplicate. The PCR efficiency and threshold cycle (Ct) were calculated using LightCycler ® 480 software. Only triplicates with a Ct standard deviation below 0.5 were kept for the following analyses. Threshold cycle values were expressed relative to two housekeeping genes (*rp49* and *tub84b*)^52^ according to the procedure described in^53^, which is based on the common method of 2^−ΔΔCt^. After normalization and inter-run calibration, we obtained the calibrated normalized relative quantity. We also measured the coefficient of variation (CV) and gene stability (M) for reference genes. We found that *rp49* and *tub84b* had a CV of 9.3% and 8.9%, respectively, which are considered as acceptable values (*i.e*., <25^53^). M was equal to 0.26, which means that our reference genes were stably expressed and our samples homogenous.

### Statistical analyses

All data are presented as mean ± SD, and the number of individuals are indicated in the figure legends. Statistical analyses were carried out using GraphPad Prism7 software, the appropriate statistical tests were applied, and only significant differences are indicated in the plots.

## Supplementary information

Supplementary materials.
