# Supplementary material for: The role of innate immunity in the protection conferred by a bacterial infection against cancer: study of an invertebrate model
Source: Sci Rep. 2020 Jun 22;10:10106. doi: 10.1038/s41598-020-66813-0 (PMC7308315; doi:10.1038/s41598-020-66813-0)
Supplement: Supplementary file 1 — Supplementary materials. [file 41598_2020_66813_MOESM1_ESM.docx]

Supplementary Materials for

The role of innate immunity in the protection conferred by a bacterial infection against cancer: study of an invertebrate model

Camille Jacqueline, Jean-Philippe Parvy, Marie-Lou Rollin, Dominique Faugère, François Renaud, Dorothée Missé, Frédéric Thomas, Benjamin Roche

**Table S1**| Prevalence of infection at day one after exposure and persistence of infection two days after exposure.

|  | Prevalence | |
| --- | --- | --- |
|  | Day 1 | Day 2 |
| *Pcc*-infected larvae | 93.3% | 37% |
| *Bb*-infected larvae | 46.7% | 56.7% |

**Table S2**| Primers used in this study

| **Gene names** | **Symbol** | **Forward (**5’-3’) | **Reverse (**5’-3’) |
| --- | --- | --- | --- |
| *diptericin* | *dpt* | GCTGCGCAATCGCTTCTACT | TGGTGGAGTGGGCTTCATG |
| *drosomycin* | *drs* | CGTGAGAACCTTTTCCAATATGATG | TCCCAGGACCACCAGCAT |
| *unpaired 3* | *upd3* | GCGGGGAGGATGTACC | GTCTTCATGGAATGAGCC |
| *ribosomal protein 49* | *rp49* | GACGCTTCAAGGGACAGTATCTG | AAACGCGGTTCTGCATGAG |
| *alpha-tubulin 84b* | *tub84b* | TGTCGCGTGTGAAACACTTC | AGCAGGCGTTTCCAATCTG |


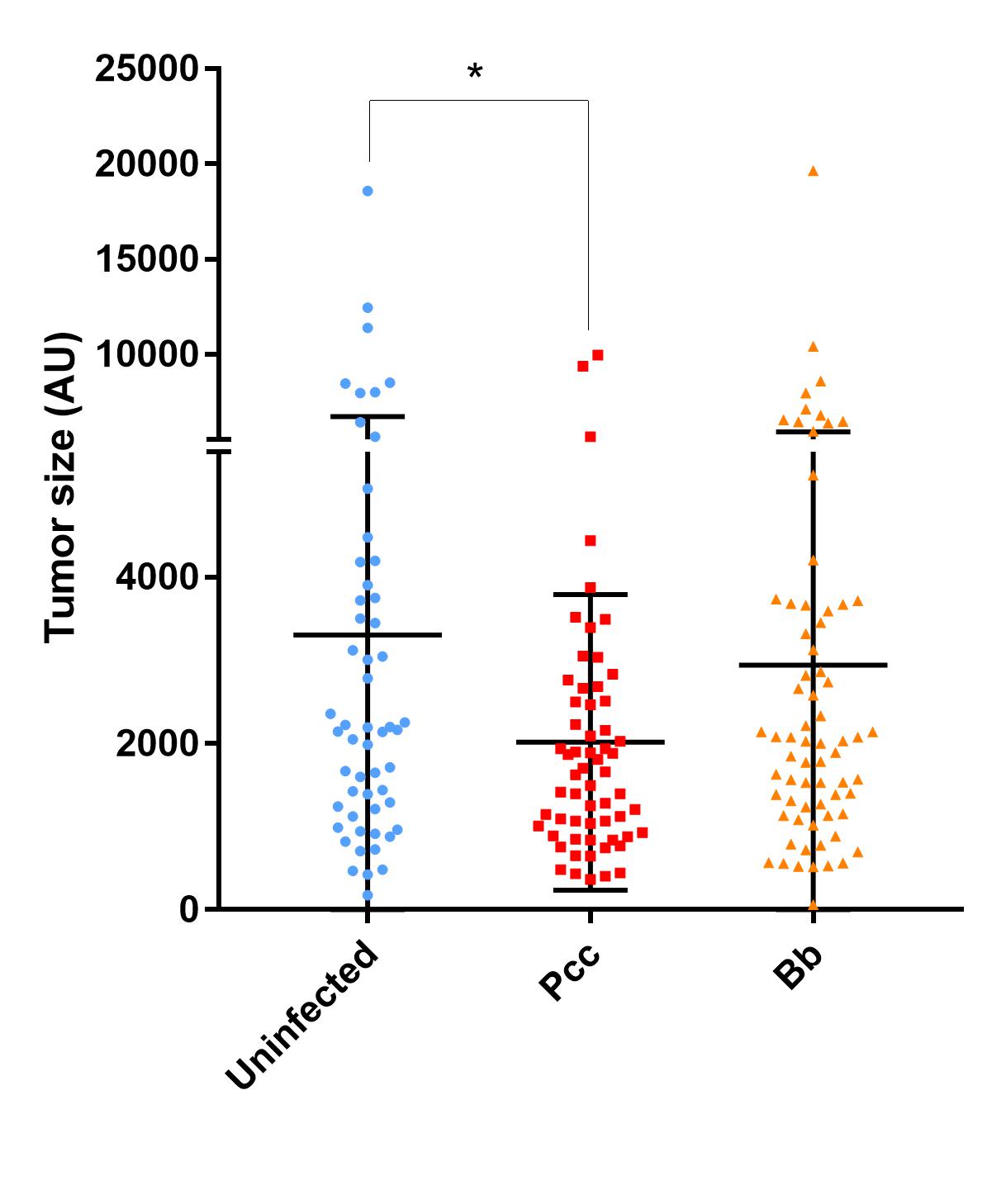


**Figure S1|** Effect of infection by bacterium *Pectobacterium carotovorum carotovorum* (n=62, abbreviated by *Pcc* in red) and the fungi *Beauvaria bassiana* (n=70, abbreviated *Bb* in orange) compared to uninfected (n=56, in blue) on individual tumor size with a high threshold of detection (S3 threshold; 90% of maximum intensity value). Data showed here were pooled from 5 independent experiments (***: p<0.001; **: p,0.01; *: p<0.05; Tukey’s test).

**
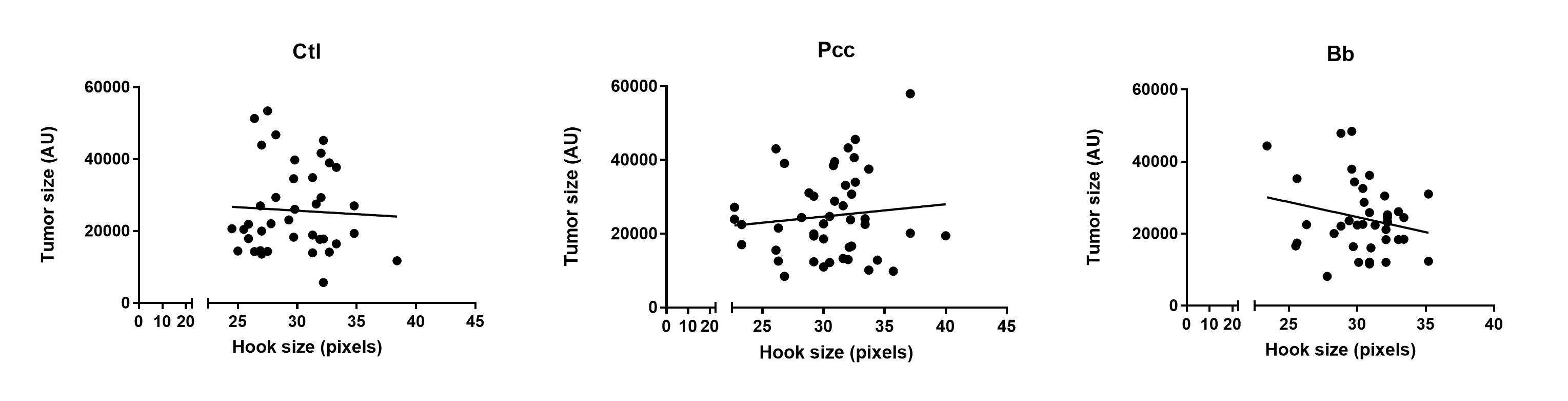
**

**Figure S2| Impact of body size on tumor size depending on infectious treatment**. Tumor size is not correlated with body size (Spearman test, p>0.05).

**Supplementary text : Tumor visualization**

Before correction, pixel intensities were comprised between 1 and 4000 (Fig. S3). Regarding image analyses, we noticed that the intensity of halogen lamp was not correlated with tumor size (see Fig. S4A) and thus we did not consider this factor in our analyses. However, we found that exposure time significantly impact tumor size estimation and thus we standardized our tumor measurements by the exposure time. This correction allowed an absence of correlation between tumor size and exposure time (Fig. S4B).

In order to control the effect of infectious treatments on tumor size through a modification of larval size, we also measured the mouth hook as a proxy of body size (Mirth et al. 2005) in ImageJ software. We looked for an effect of treatment on hook size with a specific linear model and realized pairwise comparisons of body size between groups using t-tests. Then, for each quantification of tumor size (S1 to S4), we have designed GLMMs where tumor size was used as the response variable, hook size as fixed factors and the exposure time as a random factor. We fitted each model to the most parsimonious distribution and conducted variable selection as described in the methods. The effect of body size was not significantly different depending on infectious treatment and tumor size was not affected by body size (see Fig. S5A and B).


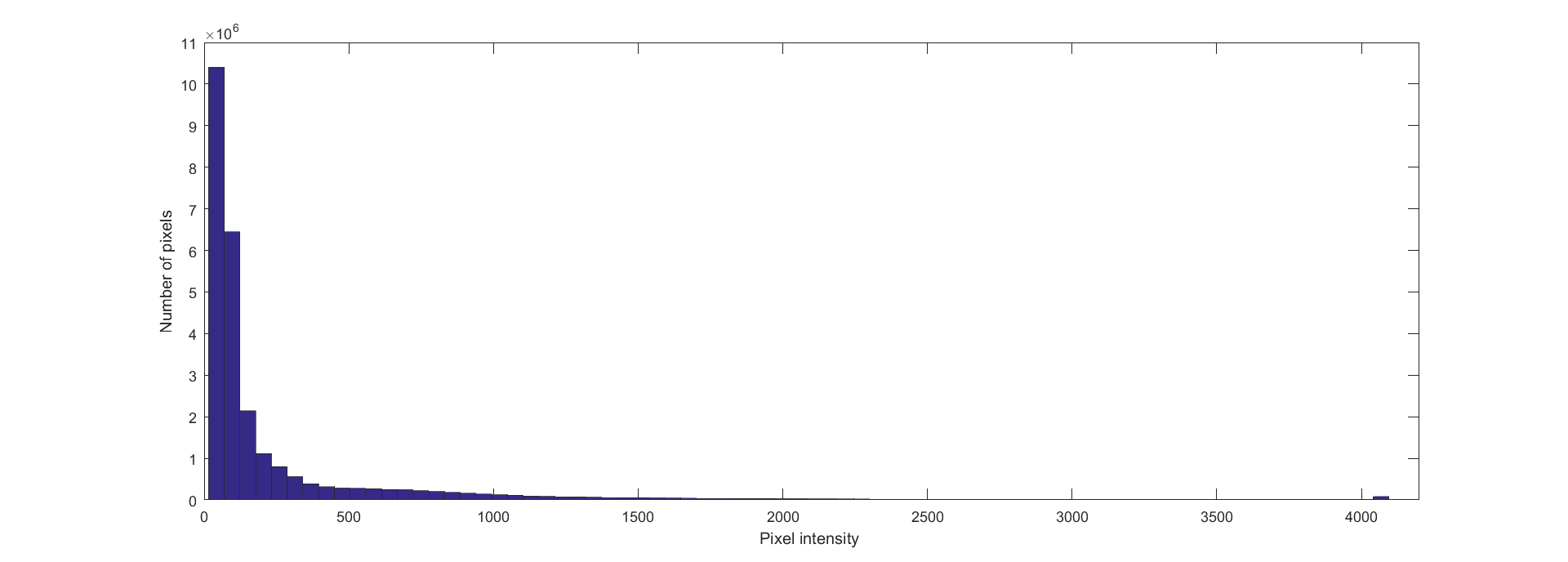


**Figure S3| Histogram of pixel intensities for each of the 759 tumor pictures**. The total number of pixels considered was above 25 billion and the figure represents data before transformation.


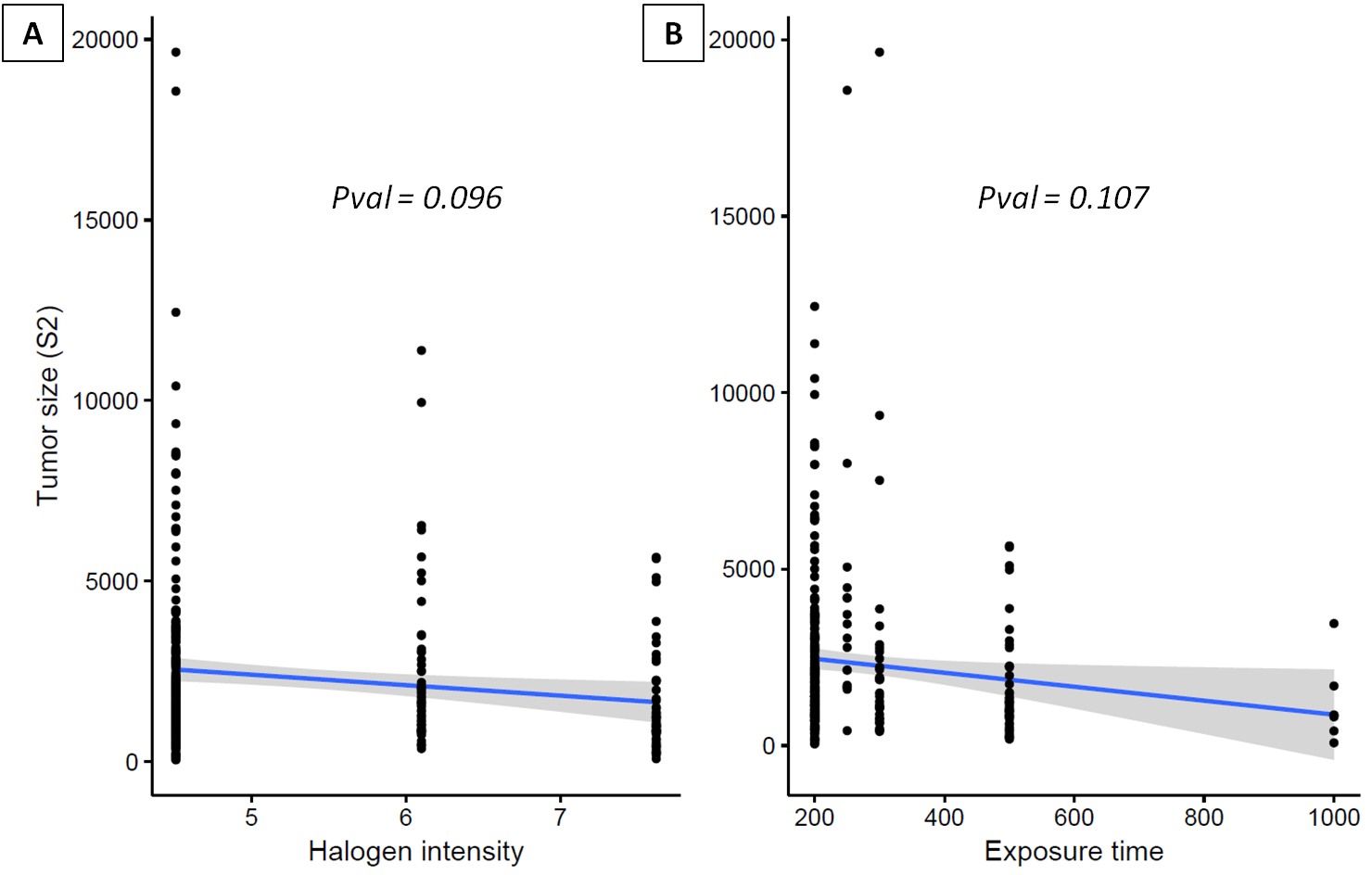


**Figure S4| Picture characteristics.** A) Absence of correlation between halogen intensity of GFP lamp and tumor size. B) Absence of correlation between exposure time and standardized tumor size.

**References:**

1. E. De Gregorio, P. T. Spellman, G. M. Rubin, B. Lemaitre, Genome-wide analysis of the Drosophila immune response by using oligonucleotide microarrays. *Proc. Natl. Acad. Sci.* **98**, 12590–12595 (2001).

2. N. Buchon, N. A. Broderick, M. Poidevin, S. Pradervand, B. Lemaitre, Drosophila Intestinal Response to Bacterial Infection: Activation of Host Defense and Stem Cell Proliferation. *Cell Host Microbe*. **5**, 200–211 (2009).

3. J. C. Pastor-Pareja, M. Wu, T. Xu, An innate immune response of blood cells to tumors and tissue damage in Drosophila. *Dis. Model. Mech.* **1**, 144–54; discussion 153 (2008).

4. J.-P. Parvy *et al.*, The antimicrobial peptide Defensin cooperates with Tumour Necrosis Factor to drive tumour cell death in Drosophila. *bioRxiv*, 513747 (2019).

5. Y. Xia, S. Shen, I. M. Verma, NF-κB, an Active Player in Human Cancers. *Cancer Immunol. Res.* **2** (2014) (available at http://cancerimmunolres.aacrjournals.org/content/2/9/823).

6. G. P. Dunn, L. J. Old, R. D. Schreiber, The three Es of cancer immunoediting. *Annu. Rev. Immunol.* **22**, 329–60 (2004).

7. E. De Gregorio, P. T. Spellman, P. Tzou, G. M. Rubin, B. Lemaitre, The Toll and Imd pathways are the major regulators of the immune response in Drosophila. *EMBO J.* **21** (2002).

8. M. Lagueux, E. Perrodou, E. A. Levashina, M. Capovilla, J. A. Hoffmann, Constitutive expression of a complement-like protein in toll and JAK gain-of-function mutants of Drosophila. *Proc. Natl. Acad. Sci. U. S. A.* **97**, 11427–32 (2000).

9. I. Kucinski, M. Dinan, G. Kolahgar, E. Piddini, Chronic activation of JNK JAK/STAT and oxidative stress signalling causes the loser cell status. *Nat. Commun.* **8**, 136 (2017).
